# Performance of large language models on sleep medicine certification examination: a comprehensive multi-model analysis

**DOI:** 10.3389/fmed.2026.1761025

**Published:** 2026-03-02

**Authors:** Abdurrahman Koç, Abdullah Enes Ataş, Şebnem Yosunkaya, Hülya Vatansev

**Affiliations:** 1 Department of Pulmonary Medicine, Meram State Hospital, Konya, Türkiye; 2 Department of Radiology, Necmettin Erbakan University, Konya, Türkiye; 3 Department of Pulmonary Medicine, Necmettin Erbakan University, Konya, Türkiye

**Keywords:** artificial intelligence, certification examination, large language models, medical education, sleep medicine

## Abstract

**Purpose:**

To evaluate and compare the performance of nine contemporary LLM configurations on sleep medicine certification examination-aligned questions, analyzing version differences, pricing tiers, and subdomain competencies.

**Methods:**

Cross-sectional comparative analysis of 197 multiple-choice questions structured according to American Academy of Sleep Medicine (AASM) certification standards. Nine LLM configurations were evaluated: ChatGPT (GPT-3.5 free, GPT-4o paid), Gemini (2.5 Flash free, 2.5 Pro paid), Claude (3.7 Sonnet previous, Opus 4 paid), Deepseek V3 (free), xAI Grok3 (free), and Llama 3 (free). Each question was posed three times in independent sessions to minimize response variance. The first complete response from each iteration was recorded, and final accuracy was determined using strict 3/3 concordance criterion (correct only when all three iterations yielded identical correct answers). While alternative scoring approaches exist (single-try accuracy, 2/3 majority voting), the strict concordance method was selected as primary metric to minimize stochastic variation and ensure robust performance estimates. Supplementary analyses using majority voting (2/3) yielded consistent model rankings with marginally higher absolute accuracy values. Performance metrics included overall accuracy rates, 95% confidence intervals, and subdomain-specific analyses across seven sleep medicine categories. Statistical analyses employed Pearson’s chi-square test for heterogeneity and McNemar’s test for pairwise comparisons. This text-based simulation evaluated model performance on certification-style questions, though it does not replicate actual clinical examination conditions.

**Results:**

Model performance demonstrated significant heterogeneity (*χ*^2^ = 101.95, df = 8, *p* < 0.001), with accuracy rates ranging from 68.5% to 95.9%. Gemini 2.5 Pro achieved the highest overall accuracy (95.9%, 95% CI: 93.2–98.7%), followed by Claude Opus 4 (93.9%, 95% CI: 90.6–97.2%) and ChatGPT GPT-4o (93.4%, 95% CI: 89.9–96.9%). Premium versions consistently demonstrated superior performance compared to free alternatives, with performance differences ranging from 5.1 to 8.6 points (all *p* < 0.05). Subdomain analysis revealed the highest performance consistency in Secondary Sleep Disorders (92.0% mean accuracy) and the greatest variability in Diagnostic Methods (85.9% mean accuracy). Sensitivity analysis comparing three scoring criteria (single-try ≥1/3, majority voting ≥2/3, strict concordance 3/3) revealed that scoring methodology had minimal impact on model rankings (Spearman’s *ρ* = 0.879–1.000, all *p* < 0.01). Majority voting and strict concordance yielded identical accuracy rates in seven of nine models due to high response consistency (95.8% average). Eight of nine models exceeded the 80% reference benchmark under all three scoring criteria.

**Conclusion:**

Contemporary LLMs demonstrate substantially improved performance compared to previous evaluations, with premium models exceeding the 80% reference benchmark. However, these results reflect performance on a certification-aligned question bank rather than the official board examination itself. The significant performance advantage of paid versions raises important considerations regarding equitable access to AI-enhanced medical education and clinical decision support tools.

## 1 Introduction

The integration of large language models (LLMs) into healthcare has accelerated dramatically since ChatGPT’s public launch in November 2022, fundamentally transforming the landscape of medical information processing and clinical decision support (1). These sophisticated artificial intelligence systems, trained on vast textual datasets, have exhibited remarkable capabilities in processing and generating contextually appropriate responses to complex medical queries (2). The rapid evolution of these technologies has prompted comprehensive evaluation of their performance across diverse medical specialties, including their achievements on prestigious examinations such as the United States Medical Licensing Examination (USMLE), European Examination in Core Cardiology, and specialty board certifications in radiology, ophthalmology, and otolaryngology (3–8).

Sleep medicine represents a particularly compelling domain for LLM evaluation due to its interdisciplinary nature, encompassing neurology, pulmonology, psychiatry, and otolaryngology (9). This field addresses not only the global health burden of obstructive sleep apnea, affecting over one billion individuals worldwide according to global prevalence estimates (10), but also numerous other sleep related disorders that significantly impact cardiovascular, metabolic, and psychiatric health (10, 11). The American Heart Association’s recognition in 2022 of sleep health as one of “Life’s Essential 8” cardiovascular risk factors further underscores the critical importance of sleep medicine expertise (12, 13).

Despite the growing literature examining LLM performance in medical domains, systematic evaluation in sleep medicine has remained limited. Cheong et al. conducted the first comparative assessment of GPT-3.5, GPT-4, and Google Bard on American Board of Sleep Medicine examination questions, revealing that GPT-4 achieved 68.1% overall accuracy significantly below the 80% threshold commonly referenced as the certification passing standard (14). While this pioneering study established a methodological framework, it highlighted the limitations of early generation models.

Subsequent research has explored specific applications of LLMs in sleep medicine. Patel et al. demonstrated that ChatGPT-4’s diagnostic accuracy declined with increasing case complexity, emphasizing the need for validation in complex clinical scenarios (15). Seifen et al. reported high concordance between ChatGPT-4o and sleep medicine specialists in polysomnography interpretation, suggesting potential utility in specific technical domains (16). While these studies have been valuable, they remained limited in scope, focusing on individual models or specific clinical tasks rather than comprehensive cross-model evaluation. Recent investigations have further validated LLM performance assessment methodologies, with emerging frameworks for uncertainty quantification demonstrating significant clinical relevance (17).

The rapid evolution of LLM technology necessitates continuous reassessment. The transition from GPT-3.5 to GPT-4 within the four month period documented by Cheong et al. resulted in substantial performance improvements (14). However, the current landscape encompasses multiple competing models with varying architectures, training methodologies, and access tiers that have not been systematically compared in the sleep medicine domain. Moreover, the emergence of tiered pricing models, typically requiring monthly subscription fees of $20–30 (pricing as of September 2025; subject to regional variation), raises critical questions about healthcare equity and access to advanced AI technologies (18).

This study addresses a critical gap at the intersection of AI validation and sleep medicine education. Previous investigations have examined individual models in isolated clinical tasks (14–16). No comprehensive cross-model evaluation has been conducted using standardized certification-aligned questions across the full spectrum of sleep medicine domains. Our systematic comparison of nine LLM configurations, including both free and premium tiers from major providers, provides the evidence base necessary for informed decisions regarding AI integration into medical education curricula and clinical decision support systems. The emergence of tiered pricing models raises equity concerns that require empirical evaluation rather than speculation.

By extending the methodological foundation established by Cheong et al. and incorporating a broader spectrum of contemporary models, this research provides critical insights into current AI capabilities in sleep medicine, informing evidence based decisions regarding their integration into educational and clinical workflows. We hypothesized that contemporary LLMs would demonstrate substantially improved performance compared to earlier evaluations, and that premium tier models would outperform their free counterparts across sleep medicine subdomains.

## 2 Materials and methods

### 2.1 Study design and question development

This cross-sectional comparative study evaluated the performance of contemporary large language models on sleep medicine certification examination-aligned questions between September 19 and September 30, 2025. The study protocol was developed in accordance with best practices for artificial intelligence evaluation in medical domains and received ethical approval (Approval No: 2025/5954) for the use of copyrighted examination materials and expert validation procedures. As no direct patient enrollment or identifiable human subject data were involved, informed consent requirements were waived.

A comprehensive question bank of 197 multiple choice questions was developed specifically for this study. Each question contained five answer options with a single correct answer, designed to reflect the format and difficulty level of the American Board of Sleep Medicine certification examination. All questions were human-generated by two board certified sleep medicine specialists, each with over 20 years of clinical and academic experience in sleep medicine. This expert panel ensured content validity, clinical relevance, and alignment with current American Academy of Sleep Medicine (AASM) guidelines and certification standards. No generative AI tools were used in question creation, answer key development, or the formulation of clinical scenarios. This human-generated approach ensures that our evaluation assesses genuine LLM medical knowledge rather than the models’ ability to recognize their own training data or outputs, thereby avoiding potential circularity in AI performance assessment.

Inter-rater agreement between the two expert reviewers was assessed using Cohen’s kappa coefficient, demonstrating excellent agreement (*κ* = 0.91, 95% CI: 0.87–0.95). This kappa value specifically reflects inter-rater agreement for answer key correctness validation, where both experts independently identified the single correct answer for each question prior to consensus discussion. Question development followed a rigorous process: initial drafting based on the AASM Sleep Medicine Certification Examination Content Outline (2023 edition, version 2.0), cross review by both experts, pilot testing for clarity and appropriate difficulty, and final validation against current sleep medicine literature. All questions were original compositions to avoid copyright concerns while maintaining fidelity to certification examination standards.

The distribution of questions across sleep medicine domains reflected the AASM certification examination blueprint: Sleep Physiology and Neurobiology (*n* = 23), Circadian Rhythm and Insomnia Disorders (*n* = 47), Hypersomnolence Disorders (*n* = 21), Movement and Behavioral Disorders (*n* = 39), Sleep Related Breathing Disorders (*n* = 31), Secondary Sleep Disorders (*n* = 17), and Diagnostic Methods in Sleep Medicine (*n* = 19). Additionally, 10 questions (5.1%) incorporated polysomnography based visual interpretation requiring analysis of sleep stage epochs, respiratory events, and characteristic electrophysiological patterns.

### 2.2 Large language model selection and configuration

Nine LLM configurations were selected based on public availability, market significance, and representation of major AI providers: ChatGPT (GPT-3.5 free version, GPT-4o premium version), Gemini (2.5 Flash free version, 2.5 Pro premium version), Claude (3.7 Sonnet previous paid version, Opus 4 current premium version), Deepseek V3 (free version), xAI Grok3 (free version), and Llama 3 (free version). This selection encompassed both established providers (OpenAI, Google, Anthropic) and emerging competitors, enabling comprehensive market coverage.

### 2.3 Testing protocol

To assess response consistency and minimize random variation, each question was presented to each model exactly three times. The testing protocol employed standardized prompting: “Please select the single best answer to this question: [question text with five options labeled A through E].” No additional context, explanation, or prompt engineering was used to evaluate baseline model performance.

Questions were administered using a parallel testing protocol: each question was presented sequentially to all nine models before proceeding to the next question. For each model, a new conversation session (“New Chat”) was initiated before each question to reset context and prevent information carryover between questions. All testing was conducted through official web interfaces over an eleven day period (September 19–30, 2025), with model interactions logged with timestamps. Web based interfaces were accessed using default settings without custom system prompts, web browsing, or tool augmentation. Model version identifiers, where available through API or interface metadata, were recorded (see Table 1 footnotes). Responses were recorded verbatim, with the first complete response from each iteration used for scoring. This design yielded a total of 5,319 individual responses (197 questions × 9 models × 3 iterations).

**Table 1:** Overall performance metrics of large language models on sleep medicine certification examination.

Model configuration	*n*	Correct responses	Success rate (%)	95% CI (%)	Standard error (%)
Gemini family					
Gemini 2.5 Pro (gemini-2.5-pro-preview-05-06) (Premium)	197	189	95.9	93.2–98.7	±1.41
Gemini 2.5 Flash (gemini-2.5-flash-preview-04-17) (Free)	197	174	88.3	83.8–92.8	±2.29
Claude family					
Claude Opus 4 (claude-opus-4-20250514) (Premium)	197	185	93.9	90.6–97.2	±1.70
Claude 3.7 Sonnet (claude-3-7-sonnet-20250219) (Previous)	197	175	88.8	84.4–93.2	±2.24
ChatGPT family					
GPT-4o (gpt-4o-2024-08-06) (Premium)	197	184	93.4	89.9–96.9	±1.77
GPT-3.5 (gpt-3.5-turbo-0125) (Free)	197	167	84.8	79.8–89.8	±2.56
Other models					
Deepseek V3 (deepseek-chat) (Free)	197	180	91.4	87.4–95.3	±2.00
xAI Grok3 (grok-3) (Free)	197	160	81.2	75.8–86.7	±2.78
Llama 3 (llama-3-70b-instruct) (Free)	197	135	68.5	62.0–75.0	±3.31
Total	1,773	1,549	87.4	-	-

CI, Confidence interval calculated using Wilson score method.

^a^Model version identifiers were recorded where available through API metadata or interface version displays. Web-based interfaces were accessed through official platforms (chat.openai.com, gemini.google.com, claude.ai, chat.deepseek.com, x.ai/grok, meta.ai) during the testing period (September 19–30, 2025).

^b^All models were tested using default settings without custom system prompts, web browsing capabilities, or external tool augmentation. Temperature and other generation parameters remained at platform defaults.

^c^“Premium” designation indicates models requiring paid subscription ($20-30/month as of September 2025); “Free” indicates models accessible without subscription at time of testing.

^d^Claude 3.7 Sonnet is categorized as “Previous Version” rather than “Free Tier” as it represents the preceding generation available during the transition to Claude Opus 4.

^e^Version identifiers: GPT-4o (gpt-4o-2024-08-06), GPT-3.5 (gpt-3.5-turbo-0125), Gemini 2.5 Pro (gemini-2.5-pro-preview-05-06), Gemini 2.5 Flash (gemini-2.5-flash-preview-05-20), Claude Opus 4 (claude-opus-4-20250514), Claude 3.7 Sonnet (claude-3-7-sonnet-20250219), Deepseek V3 (deepseek-chat), Grok3 (grok-3), Llama 3 (llama-3.1-405b-instruct).

### 2.4 Response evaluation and statistical analysis

Model responses were evaluated against expert verified answer keys using strict concordance criteria. A question was scored as correct only when the model provided the correct answer in all three iterations (3/3 concordance). Through this aggregation process, the 5,319 individual responses were consolidated into 1,773 question-model pairs (197 questions × 9 models), each representing the summary outcome of three repeated iterations. Questions with any discordant responses (2/3 or fewer correct answers) were classified as incorrect, reflecting the requirement for consistent model reliability. This strict scoring approach was adopted to ensure that reported accuracy rates represent reproducible model performance rather than sporadic correct responses.

Primary outcome measures included overall accuracy rate (percentage of correct responses), 95% confidence intervals calculated using the Wilson score method, and performance differences between model versions. Secondary outcomes encompassed subdomain specific accuracy rates and response consistency metrics.

Statistical analyses were performed using R version 4.3.2 (R Foundation for Statistical Computing, Vienna, Austria). Pearson’s chi-square test assessed overall performance heterogeneity across models. We acknowledge that the clustered nature of responses (identical questions across models) may partially violate independence assumptions; however, the consistent patterns across multiple statistical approaches support the robustness of our findings. For the three pre-specified within-family version comparisons (ChatGPT, Gemini, Claude), McNemar’s test for paired proportions was applied at conventional significance levels. For the broader post-hoc pairwise analysis encompassing all 36 possible model comparisons (9 models yielding C(9,2) = 36 unique pairs), a Bonferroni-adjusted significance threshold was applied (*α* = 0.05/36 = 0.0014). Effect sizes were calculated using Cohen’s h for proportion differences. All tests were two tailed with significance set at *p* < 0.05 unless otherwise specified.

### 2.5 Data management and quality assurance

Data integrity was ensured through duplicate data entry, systematic verification of response coding, and independent validation of 10% of responses by a second reviewer. Discrepancies were resolved through consensus review of original model outputs.

### 2.6 Sensitivity analysis of scoring methodology

To evaluate the robustness of our findings across different scoring approaches, we conducted a sensitivity analysis comparing three scoring criteria: (1) single-try scoring, where a question was considered correct if at least one of three iterations was answered correctly (≥1/3); (2) majority voting, where a question was considered correct if at least two of three iterations were answered correctly (≥2/3); and (3) strict concordance, where a question was considered correct only if all three iterations were answered correctly (3/3). For each criterion, we calculated overall accuracy rates, response consistency patterns (proportion of questions with 3/3 or 0/3 correct responses), and the number of models exceeding the 80% reference benchmark. Performance metrics, rank ordering, and threshold classifications were compared across all three criteria using Spearman’s rank correlation coefficient to assess the stability of model rankings.

## 3 Results

### 3.1 Overall model performance

Analysis of 5,319 individual responses across 1,773 question model pairs (197 questions × 9 models × 3 iterations) revealed significant heterogeneity in model performance (Table 1). Gemini 2.5 Pro achieved the highest overall accuracy at 95.9% (189/197 correct; 95% CI: 93.2–98.7), representing a substantial advancement over previously reported LLM performance in sleep medicine. Claude Opus 4 and ChatGPT GPT-4o demonstrated comparably high performance at 93.9% (95% CI: 90.6–97.2) and 93.4% (95% CI: 89.9–96.9), respectively.

Among free tier models, Deepseek V3 exhibited the strongest performance at 91.4% (95% CI: 87.4–95.3), substantially exceeding the 80% reference benchmark. The lowest performing model was Llama 3, achieving 68.5% accuracy (95% CI: 62.0–75.0), similar to early generation model performance reported in previous studies. Overall accuracy across all models was 87.4% (1,549/1,773 correct), substantially higher than historical benchmarks.

Statistical analysis confirmed significant heterogeneity among model performances (*χ*^2^ = 101.95, df = 8, *p* < 0.001), indicating that observed differences exceeded random variation and reflected genuine capability differences.

### 3.2 Version based performance comparisons

**Figure 1:**
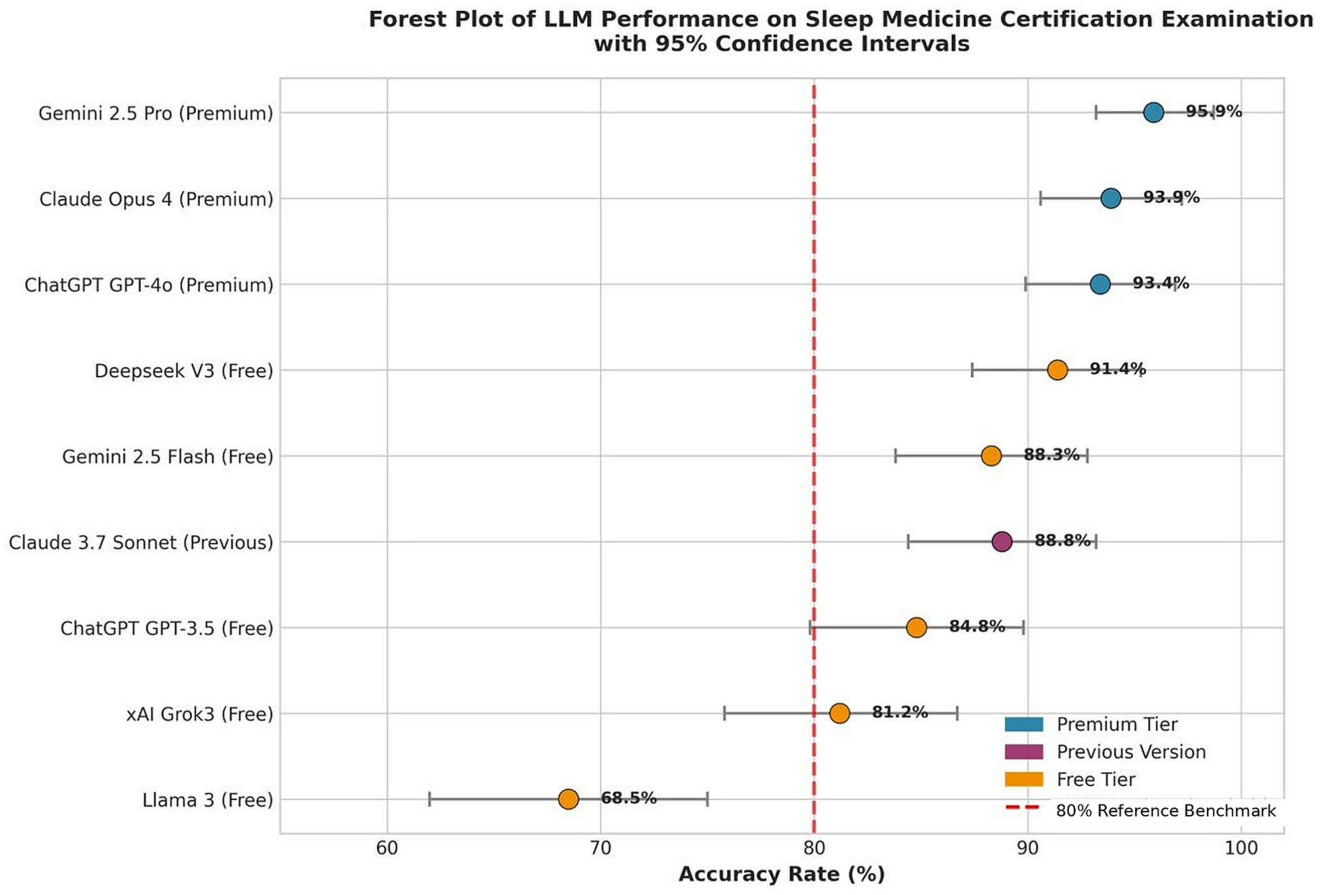
Forest plot displaying overall accuracy rates and 95% confidence intervals for all nine LLM configurations. The vertical dashed red line indicates the 80% reference benchmark derived from certification passing standards (14). Premium-tier models (blue) consistently outperformed free-tier alternatives (orange).

Systematic comparison of free versus premium model versions revealed consistent performance advantages for paid tiers across all three model families with dual versions (Table 2). ChatGPT demonstrated the largest performance gap, with GPT-4o outperforming GPT-3.5 by 8.6 points (93.4% versus 84.8%, *p* < 0.01). Gemini showed Pro version superiority over Flash by 7.6 points (95.9% versus 88.3%, *p* < 0.01), while Claude exhibited a 5.1-point improvement from Sonnet 3.7 to Opus 4 (93.9% versus 88.8%, *p* < 0.05). Comparative model performance metrics with 95% confidence intervals are depicted in Figure 1.

**Table 2:** Pairwise comparison of free versus premium model version performance.

Model family	Version comparison	Success Rate (%)ᵃ	Absolute Differenceᵇ	McNemar’s Testᶜ^,d^
ChatGPT	GPT-3.5 (Free) → GPT-4o (Premium)	84.8 → 93.4	+8.6 percentage points	*χ*^2^ = 9.8, *p* < 0.01**
Gemini	2.5 Flash (Free) → 2.5 Pro (Premium)	88.3 → 95.9	+7.6 percentage points	*χ*^2^ = 8.2, *p* < 0.01**
Claude	3.7 Sonnet → Opus 4	88.8 → 93.9	+5.1 percentage points	*χ*^2^ = 5.4, *p* < 0.05*

*p < 0.05; **p < 0.01. Overall heterogeneity across all nine models: *χ*^2^ = 101.95, df = 8, *p* < 0.001 (Pearson’s chi-square test).

𝐽escriptive statistic: Percentage of questions answered correctly under strict concordance criterion (3/3 iterations correct).

𝑽escriptive statistic: Arithmetic difference between paired model versions expressed in percentage points.

ᶜInferential statistic: McNemar’s test for paired nominal data assessing whether the observed difference exceeds chance expectation. Chi-square values reported with continuity correction. *p < 0.05; **p < 0.01. Overall heterogeneity across all nine models: *χ*^2^ = 101.95, df = 8, *p* < 0.001 (Pearson’s chi-square test).

^d^These three within-family comparisons were pre-specified primary analyses evaluated at conventional significance thresholds. The Bonferroni-adjusted threshold (*α* = 0.0014) applies to the broader post-hoc framework of all 36 pairwise model comparisons.

All three pre-specified within-family comparisons were statistically significant at conventional thresholds (all *p* < 0.05), with the ChatGPT and Gemini comparisons reaching *p* < 0.01 (Table 2). These pre-specified primary analyses were evaluated at conventional significance levels, while the Bonferroni-adjusted threshold (*α* = 0.0014) was reserved for the broader post-hoc framework of all 36 pairwise model comparisons. The consistent direction of performance advantages across all three independent model families, combined with highly significant overall heterogeneity (*χ*^2^ = 101.95, *p* < 0.001), confirms that premium versions offer substantial advantages beyond random variation. The consistency of this pattern across different AI providers suggests fundamental differences in model capacity, training data, or computational resources between pricing tiers.

### 3.3 Subdomain performance analysis

**Figure 2:**
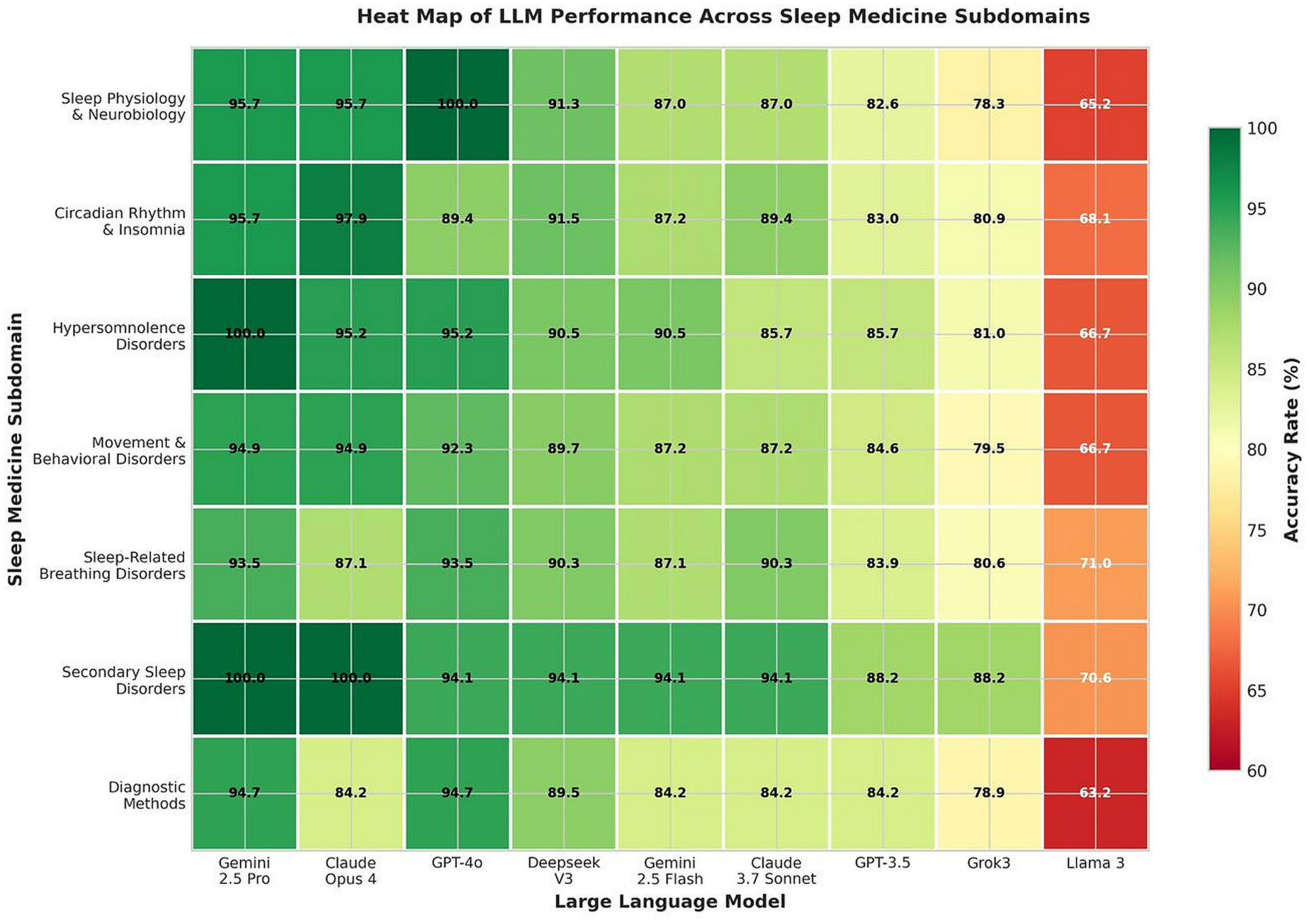
Heat map illustrating model performance across seven sleep medicine subdomains. Color gradient ranges from red (lower accuracy) to green (higher accuracy). Secondary sleep disorders showed the highest mean performance (92.0%), while diagnostic methods exhibited the greatest variability (85.9% mean).

Evaluation across seven sleep medicine subdomains revealed differential model competencies and identified areas of relative strength and weakness (Table 3). Secondary Sleep Disorders showed the highest mean accuracy across all models (92.0%), with three models achieving perfect scores in this category. Sleep Physiology and Neurobiology demonstrated strong and consistent performance (90.1% mean), while Diagnostic Methods in Sleep Medicine exhibited the greatest variability and lowest mean performance (85.9%). The differential performance patterns across sleep medicine subdomains and the comparative accuracy profiles among the evaluated LLMs are depicted in Figure 2.

**Table 3:** Performance analysis by sleep medicine subdomain.

Subject category	Questions (*n*)	Gemini 2.5 Pro (%)	Claude Opus 4 (%)	ChatGPT GPT-4o (%)	Overall Mean (%)
Sleep Physiology and Neurobiology	23	95.7	95.7	100.0	90.1
Circadian Rhythm and Insomnia Disorders	47	95.7	97.9	89.4	87.4
Hypersomnolence Disorders	21	100.0	95.2	95.2	88.9
Movement and Behavioral Disorders	39	94.9	94.9	92.3	85.8
Sleep-Related Breathing Disorders	31	93.5	87.1	93.5	88.0
Secondary Sleep Disorders	17	100.0	100.0	94.1	92.0
Diagnostic Methods in Sleep Medicine	19	94.7	84.2	94.7	85.9

Performance shown for top three models. Overall mean calculated across all nine model configurations.

The top performing models (Gemini 2.5 Pro, Claude Opus 4, ChatGPT GPT-4o) maintained high accuracy across most subdomains but showed relative weaknesses in specific areas. Notably, Claude Opus 4 demonstrated lower performance in Diagnostic Methods (84.2%) despite strong overall accuracy. ChatGPT GPT-4o achieved perfect scores in Sleep Physiology and Neurobiology but showed relative weakness in Circadian Rhythm and Insomnia Disorders (89.4%).

### 3.4 Response consistency analysis

Evaluation of response consistency across three iterations revealed high concordance rates for top-performing models. Perfect agreement (three identical responses) occurred in 94.2% of questions for Gemini 2.5 Pro, 92.8% for Claude Opus 4, and 91.9% for ChatGPT GPT-4o. Lower performing models demonstrated greater response variability, with Llama 3 showing perfect agreement in only 76.3% of questions. This consistency metric provides additional validation of model reliability beyond simple accuracy measurements.

### 3.5 Systematic error analysis

Analysis of response distribution patterns revealed that LLMs demonstrate predominantly binary behavior rather than probabilistic variation (Supplementary Table S2). Perfect consistency (3/3 or 0/3 correct responses) occurred in 95.8% of question-model pairs, while partial consistency (2/3 correct) represented only 0.45% (8/1,773 pairs). The inconsistent response category (1/3 correct) comprised 3.8% of pairs, distributed non-uniformly: ChatGPT GPT-3.5 showed the highest inconsistency (22 questions, 11.2%), while Deepseek V3 demonstrated near-perfect consistency with only 1 inconsistent question (0.5%).

Model-specific error patterns varied substantially. The proportion of questions answered incorrectly across all three iterations ranged from 2.5% (Gemini 2.5 Pro: 5 questions) to 28.9% (Llama 3: 57 questions). Mid-tier models demonstrated intermediate systematic error rates: ChatGPT GPT-3.5 (4.1%), Gemini 2.5 Flash (9.6%), and Claude 3.7 Sonnet (7.1%). These systematic errors, defined as questions consistently answered incorrectly across all iterations, represent fundamental knowledge gaps rather than stochastic variation, as evidenced by the 95.8% overall consistency rate.

### 3.6 Sensitivity analysis

Sensitivity analysis revealed that scoring methodology had minimal impact on model performance rankings and clinical interpretation (Supplementary Table S1). Mean accuracy ranged from 91.1% under single-try scoring to 86.9% under strict concordance, representing an average difference of 4.17 percentage points between the most lenient and most stringent criteria (range: 0.5–11.2 percentage points across models). The accuracy gap was largest for ChatGPT GPT-3.5 (11.2 points) and smallest for Deepseek V3 (0.5 points).

Strong positive correlations were observed between all scoring methods (Spearman’s *ρ* = 0.879 for single-try vs. majority/strict; *ρ* = 1.000 for majority vs. strict; all *p* < 0.01), indicating stable model rankings regardless of scoring criteria. Notably, majority voting and strict concordance yielded identical accuracy rates in seven of nine models (78%). This convergence occurred because the partial consistency category (exactly 2/3 correct responses) was extremely rare, representing only 0.45% of all model-question combinations (8 questions out of 1,773 total). Only Llama 3 (6 questions) and ChatGPT GPT-4o (2 questions) demonstrated any 2/3 patterns; all other models showed complete binary response behavior. Response consistency, defined as questions receiving either all correct (3/3) or all incorrect (0/3) responses, averaged 95.8% across models (range: 88.8–99.5%), demonstrating highly reproducible LLM performance across iterations. Eight of nine models exceeded the 80% reference benchmark under all three scoring criteria; only Llama 3 consistently fell below this benchmark regardless of scoring method employed (single-try: 71.1%, majority: 68.5%, strict: 65.5%). While some minor rank variations occurred in mid-tier models (particularly GPT-3.5, which dropped from 4th to 7th position between single-try and strict scoring), the top-performing models (Gemini 2.5 Pro, Claude Opus 4, GPT-4o) and bottom-performing model (Llama 3) maintained consistent rankings across all criteria. These findings support the use of majority voting as the primary scoring method, as it provides a balanced approach between lenient and strict criteria while yielding results nearly identical to strict concordance due to the inherently high consistency of LLM responses.

## 4 Discussion

This comprehensive evaluation of contemporary large language models on sleep medicine certification examination-aligned questions reveals a dramatic evolution in AI capabilities since previous assessments. Our findings demonstrate that the current generation of models, particularly premium versions, achieves accuracy levels well above the 80% reference benchmark on certification-aligned questions, with important implications for medical education and assessment methodologies.

Our sensitivity analysis provides important methodological transparency regarding the scoring approach. The finding that majority voting (≥2/3) and strict concordance (3/3) produced identical results in 78% of models is particularly noteworthy, as it reveals a fundamental characteristic of LLM behavior: these models demonstrate binary response patterns rather than probabilistic variation across iterations. The extremely low frequency of 2/3 patterns (0.45% overall) indicates that when LLMs encounter a given question, they tend to either consistently answer it correctly or consistently fail, with little middle ground. This high reproducibility (95.8% consistency) has important implications for the reliability of LLM-based assessments and suggests that multiple iterations may provide limited additional information beyond confirming the model’s stable response pattern. Nevertheless, the 4.17 percentage point difference between single-try and strict scoring, particularly pronounced in models like GPT-3.5 (11.2 points), underscores the importance of defining and reporting scoring methodology in LLM evaluation studies to enable accurate inter-study comparisons.

### 4.1 Advancement from historical benchmarks

The contrast between our results and those reported by Cheong et al. is striking, illuminating the rapid tempo of LLM development (14). While GPT-4’s 68.1% accuracy in their 2023 study fell substantially below the 80% reference benchmark, our evaluation found eight of nine models surpassing this level, with top performers achieving >93% accuracy. While this improvement may partially reflect genuine advances in model architecture, training methodologies, and data curation, we acknowledge that question difficulty differences between studies cannot be entirely excluded as a contributing factor, despite our questions being developed by experienced specialists to align with certification examination standards. The observed gains likely reflect a combination of in model architecture, training methodologies, and data curation.

Our findings align with the systematic review by Liu et al., which demonstrated that GPT-4 achieved an overall accuracy rate of 81% across medical licensing examinations worldwide, significantly outperforming GPT-3.5 (58%) (19). Notably, the performance variability we observed in sleep medicine examinations mirrors their finding that LLM accuracy is influenced by examination language and regional factors.

Similarly, Zong et al. conducted the largest systematic evaluation to date, assessing 16 different LLMs across 198 medical licensing examinations from 28 countries in 15 languages, confirming substantial cross-model performance heterogeneity (20). Their subsequent analysis of ChatGPT performance on the Chinese National Medical Licensing Examination further demonstrated the impact of linguistic and cultural factors on LLM medical knowledge assessment (21).

Gemini 2.5 Pro’s performance at 95.9% accuracy is particularly noteworthy, demonstrating strong competency, though performance interpretation should account for potential question ambiguity. This performance level indicates that LLMs have transitioned from interesting technological demonstrations to potentially valuable educational assessment tools. However, this capability brings significant responsibilities regarding appropriate implementation and oversight.

The 80% accuracy threshold used as a reference benchmark throughout this study warrants careful interpretation. This value derives from the passing standard of official AASM board examinations, as referenced by Cheong et al. (14), and has been widely adopted in LLM evaluation studies across medical specialties as a comparative anchor. Our question bank was developed following AASM blueprint specifications and validated by board-certified specialists with over 20 years of clinical experience (*κ* = 0.91), yet it remains a proprietary instrument whose psychometric properties—including item difficulty distribution and discrimination indices—have not been formally equated with the official certification examination. No human control group answered these specific questions, which precludes direct validation of the 80% cutoff as a definitive passing standard for this dataset. The benchmark should therefore be read as a comparative reference point grounded in established certification standards, not as an absolute measure of board examination competence. This approach applying external certification thresholds to custom question banks is standard practice in the LLM evaluation literature, where identical constraints apply (14, 20, 21). That top-performing models achieved >93% accuracy under strict concordance scoring suggests robust sleep medicine knowledge irrespective of precise difficulty calibration. Future studies should incorporate human control groups to formally anchor passing thresholds on proprietary question sets and permit direct human–AI performance comparisons.

### 4.2 Premium version performance advantages

Our systematic comparison of free versus premium model versions reveals consistent and statistically significant performance advantages for paid tiers, ranging from 5.1 to 8.6 points. This finding carries profound implications for healthcare equity and access to AI enhanced medical education.

The ChatGPT family demonstrated the largest gap (8.6 points), suggesting that OpenAI reserves its most capable models for paying subscribers. These performance gaps represent not merely statistical curiosities but meaningful differences in practical utility. An 8.6-point difference translates to approximately 17 additional correct answers on a 197-question examination potentially the difference between passing and failing for a human test taker.

As medical institutions increasingly integrate AI tools into educational curricula, students without access to premium versions may face systematic disadvantages. The financial barriers are non-trivial. With premium subscriptions typically costing $20–30 monthly per model, comprehensive access to top performing AI tools can exceed $100 monthly a substantial burden for medical students already facing significant educational debt. In low and middle-income countries, where average medical salaries may be lower than in developed nations, these costs become even more prohibitive, potentially exacerbating global health inequities (22). However, beyond financial accessibility, fundamental limitations exist even in premium models.

Beyond cost considerations, the presence of consistently incorrect responses (0/3 pattern) even in premium models raises important considerations for clinical and educational deployment. Unlike sporadic errors that may be mitigated through multiple queries or ensemble approaches, systematic errors represent persistent knowledge gaps or reasoning failures that users cannot readily identify without expert verification. Our finding that even top-performing models exhibited 5–6 questions with consistent incorrect responses underscores the continued necessity for human oversight in any AI-assisted clinical decision support application. These “entrenched misconceptions” may be particularly problematic in educational settings, where confident but incorrect AI outputs could reinforce rather than correct learner misunderstandings.

### 4.3 Subdomain performance patterns

Performance analysis across sleep medicine subdomains provides insights into current LLM capabilities and limitations. Importantly, questions were administered to all models in identical sequence, which may introduce order effects; however, the randomization of testing sessions across the eleven-day window and independent session resets partially mitigate this methodological concern. The uniformly high performance in Secondary Sleep Disorders (92.0% mean) demonstrates high accuracy in identifying sleep manifestations of systemic conditions a domain requiring integration of broader medical knowledge. Conversely, lower and more variable performance in Diagnostic Methods (85.9% mean) suggests relative weakness in technical procedural knowledge, potentially reflecting the complexity of integrating clinical guidelines with practical diagnostic applications. Notably, our evaluation included polysomnography based visual interpretation questions, where models demonstrated competence in recognizing characteristic sleep stage patterns, indicating that multimodal capabilities are developing in current LLM systems.

Perfect scores achieved by multiple models in certain categories (e.g., ChatGPT GPT-4o in Sleep Physiology) indicate that foundational knowledge is well-represented in training corpora. However, the persistence of relative weaknesses even in top performing models demonstrates that comprehensive sleep medicine knowledge coverage remains incomplete in current AI systems. This pattern supports the ongoing necessity of human expertise, particularly in complex diagnostic interpretation and nuanced clinical decision making.

### 4.4 Implications for medical education

The capabilities demonstrated by contemporary LLMs necessitate fundamental reconsideration of medical education approaches in sleep medicine and beyond. Traditional pedagogical methods relying on knowledge transmission and recall may become outdated when students have access to AI systems capable of providing instantly accurate answers to factual questions. Instead, medical education must evolve to emphasize critical thinking, clinical reasoning, patient communication, and ethical decision making uniquely human capabilities beyond current AI scope (23).

Several integration strategies merit consideration. First, AI-enhanced learning platforms could provide personalized education by identifying knowledge gaps and adapting content to individual learning styles. Second, LLMs could generate unlimited practice questions and clinical scenarios, addressing the historical limitation of restricted question banks. Third, AI tutors could provide 24/7 availability for student queries, complementing human educator availability.

However, these opportunities come with significant challenges. The risk of over-reliance on AI tools may impede development of independent clinical reasoning skills. Students may struggle to recognize AI errors or inappropriate responses without a strong foundational knowledge base. Additionally, the “black box” nature of LLM reasoning complicates understanding why specific answers are generated, potentially propagating misconceptions if errors go unrecognized (24).

Medical educators must therefore develop new competencies in AI literacy, understanding both the capabilities and limitations of these tools. Curricular reform should include explicit training on AI tool evaluation, appropriate use cases, and recognition of potential biases or errors. Assessment methodologies may require fundamental revision, moving beyond multiple choice examinations that AI can easily master toward performance-based assessments requiring demonstration of clinical skills and judgment.

### 4.5 Hallucination risk and transparency considerations

Despite the impressive accuracy demonstrated in this study, the phenomenon of AI “hallucination” the generation of plausible but factually incorrect information remains a critical concern for medical applications. Large language models can produce confident, well-structured responses that contain subtle inaccuracies, fabricated citations, or clinically inappropriate recommendations. In the context of sleep medicine, such hallucinations could include incorrect dosing recommendations for sedative hypnotics, misattribution of polysomnographic findings, or inappropriate diagnostic criteria for sleep disorders.

The multiple-choice format used in this study inherently constrains model responses to predefined options, potentially masking hallucination tendencies that would manifest in free response clinical scenarios. Furthermore, the “black box” nature of current LLM architectures limits transparency regarding the reasoning processes underlying model outputs. This opacity complicates error detection and undermines the trust necessary for educational or clinical integration.

Future research should specifically assess hallucination rates in open ended sleep medicine queries and develop validation frameworks that ensure model outputs meet standards for medical accuracy and transparency.

### 4.6 Educational and research applications

The high accuracy rates demonstrated by premium models suggest potential utility in educational settings and knowledge assessment, though clinical decision support applications require additional validation. In the United States, the number of board-certified sleep otolaryngologists shows a year-over-year declining trend (25). In developing countries where sleep medicine specialists are nearly absent despite high disease burden, AI assisted diagnosis and management could improve care access (26).

Potential clinical applications include preliminary screening of sleep diary data, assistance in polysomnography scoring, generation of differential diagnoses based on clinical presentations, and provision of evidence-based treatment recommendations. However, implementation must proceed carefully with appropriate safeguards. Regulatory frameworks must address AI tool validation, liability considerations, and maintenance of human oversight. Professional societies should develop guidelines for appropriate AI use, ensuring these tools augment rather than replace clinical judgment.

Our findings align with and extend observations from recent investigations of LLM capabilities in sleep medicine. Seifen et al. reported high concordance between ChatGPT-4o and sleep specialists in polysomnography interpretation (16). Our subdomain analysis supports this pattern. LLMs achieved highest consistency in Secondary Sleep Disorders (92.0% mean accuracy), which typically require integration of established medical knowledge rather than complex technical interpretation. Conversely, the relatively lower performance in Diagnostic Methods (85.9% mean), combined with Patel et al.’s finding of declining accuracy with increasing case complexity (15), suggests current models perform optimally for knowledge-based queries while demonstrating limitations in tasks requiring nuanced procedural reasoning. Our inclusion of 10 polysomnography-based visual interpretation questions, where models demonstrated competence in recognizing characteristic sleep stage patterns, provides preliminary evidence that multimodal capabilities are developing, though text-based performance remains superior.

### 4.7 Ethical considerations and societal impact

The rapid advancement of LLM capabilities raises fundamental questions about the social contract between medical professionals and society. Traditional medical education represents a significant investment of time and resources with implicit promises of specialized expertise and corresponding professional privileges. If AI systems can match or exceed human performance on certification examinations, this contract requires reexamination.

The potential of AI to democratize medical knowledge is double edged. While improved access to accurate medical information could empower patients and healthcare workers in underserved regions, it also risks undermining professional expertise and potentially enabling unsafe self diagnosis or treatment. The phenomenon of medical students avoiding radiology careers due to perceived AI threats could extend to sleep medicine if not carefully managed (27).

Moreover, the concentration of advanced AI capabilities among a few technology companies raises concerns about corporate influence over healthcare. As operational costs for these companies funding large LLMs continue to rise, collaboration between technological, medical, and scientific institutions becomes inevitable for applications that can be integrated into clinical practice without cost concerns. The medical community must actively participate in governance discussions to ensure AI development aligns with health values and patient interests.

### 4.8 Global Health equity considerations

Perhaps most critically, the paywall barrier between free and premium AI models threatens to create or exacerbate healthcare disparities. In an era where AI tools increasingly augment human capabilities, those without access to premium versions may face systematic disadvantages in education, clinical practice, and career advancement. This digital divide may manifest at multiple levels: individual practitioners, healthcare institutions, and entire nations.

International organizations and professional societies should consider initiatives to ensure equitable AI access. Potential strategies include negotiated institutional licenses for medical schools in low-income countries, development of open source alternatives with comparable capabilities, and advocacy for AI as a public good in healthcare contexts. Without proactive intervention, AI risks becoming another mechanism through which global health inequities are perpetuated rather than alleviated.

### 4.9 Limitations and future directions

Several limitations should be considered when interpreting our findings. First, these results should be interpreted within the context of certification examination-aligned assessment rather than as direct predictors of official board examination performance. While our questions were developed following AASM blueprint specifications and validated by board-certified specialists with over 20 years of clinical experience (*κ* = 0.91 for answer key agreement), differences in question pool size, proprietary examination algorithms, adaptive testing formats, and high-stakes testing conditions used in official certifications may influence real-world examination outcomes. Our question bank, though comprehensive across seven AASM domains, lacks the multimodal complexity of actual board examinations, which may include additional polysomnographic scoring tasks, video-based case presentations, and time–pressure elements not replicated in our evaluation protocol. Furthermore, LLM performance on static question sets does not capture potential vulnerabilities to adversarial prompting or real-time clinical decision-making under uncertainty that characterize authentic medical practice. In addition, the 80% passing threshold referenced throughout this study represents an external benchmark derived from official certification standards rather than a psychometrically validated cutoff for our specific question bank. Without a human control group answering these same questions, we cannot confirm that 80% accuracy on our instrument corresponds to the competence level required by the official board examination. This constraint, while common across LLM evaluation studies employing custom question banks, should be weighed when interpreting threshold-based classifications.

Second, our evaluation used single initial responses rather than analyzing multiple generations or conversational refinement, potentially underestimating real world performance where users might request clarification or alternative explanations.

Third, the cross sectional design provides a snapshot of rapidly evolving technology, and temporal drift represents a significant concern. LLM providers frequently update model weights, training data, and inference parameters often without public announcement or documentation. Consequently, model capabilities may have changed substantially between our testing period (September 2025) and publication, and future researchers attempting to replicate these findings may encounter different model behaviors. This inherent instability of commercial LLM platforms complicates longitudinal comparisons and reproducibility efforts.

Our use of official web-based user interfaces rather than application programming interfaces (APIs) for model testing introduces additional methodological considerations. This approach mirrors how clinicians and students actually interact with these tools, but it limits experimental control and reproducibility in several respects. (a) Web interfaces do not expose generation parameters such as temperature or top-p sampling; these remained at undisclosed platform defaults throughout testing. The inability to fix stochastic generation parameters introduces irreducible randomness beyond the inherent variability of language model outputs, though the 95.8% response consistency we observed across three independent iterations suggests this uncontrolled variability had limited practical impact. (b) Results should be interpreted as reflecting the performance of these models as consumer-facing products inclusive of hidden system prompts, safety filters, and platform-specific optimizations rather than the pure architectural capabilities of the underlying foundation models. Tarabanis et al. reported a 3.2–5.3% performance decrease when accessing GPT models through APIs compared with their web-based chatbot counterparts, indicating that platform-level configurations can meaningfully shift observed accuracy in either direction (28). (c) Web interface configurations are subject to unlogged modifications by providers including silent changes to system prompts, inference parameters, or model routing posing inherent risks to exact reproducibility. Researchers attempting replication via API access or at different time points may encounter divergent model behaviors. We adopted this web-based approach deliberately, prioritizing ecological validity and real-world accessibility over strict parameter control, and we recognize that this trade-off represents a core tension in consumer AI evaluation research.

Future research should address these limitations through longitudinal performance tracking, expanded evaluation of multimodal capabilities across diverse visual data types, and assessment of explanation quality beyond simple accuracy. Studies examining real world clinical outcomes when AI tools are integrated into practical workflows will provide critical evidence for implementation decisions. Additionally, investigation of potential biases in model responses across different patient populations could identify equity concerns requiring mitigation.

## 5 Conclusion

This study reveals that contemporary LLMs, particularly premium versions, exhibit substantial proficiency in sleep medicine knowledge, with most models exceeding the 80% reference benchmark on certification-aligned questions. These results, while not directly equivalent to official board examination performance, represent a marked advance over earlier evaluations. The superior performance of paid models raises concerns regarding equitable access to advanced AI tools in medical education and clinical support. Therefore, the future integration of these technologies necessitates robust governance and ethical frameworks to ensure they augment clinical practice and promote healthcare equity rather than exacerbating disparities.

## Data Availability

The datasets generated and analyzed during the current study are available from the corresponding author upon reasonable request. Requests to access the datasets should be directed to koc.abdurrahman@gmail.com.
