# Supplementary material for: Performance of large language models on sleep medicine certification examination: a comprehensive multi-model analysis
Source: Front Med (Lausanne). 2026 Mar 2;13:1761025. doi: 10.3389/fmed.2026.1761025 (PMC12989592; doi:10.3389/fmed.2026.1761025)
Supplement: Supplementary file 1 [file Table_1.docx]

Supplementary Material

# Supplementary Table S1. Distribution of Board Examination Questions by Sleep Medicine Domain

The distribution of questions across sleep medicine domains reflected the AASM certification examination blueprint. A total of 197 multiple-choice questions were categorized across seven core domains.

| **Domain** | **Question Count (n)** | **Percentage (%)** | **Mean Questions per Exam** |
| --- | --- | --- | --- |
| Circadian Rhythm and Insomnia Disorders | 47 | 23.9 | 5.9 |
| Movement and Behavioral Disorders | 39 | 19.8 | 4.9 |
| Sleep-Related Breathing Disorders | 31 | 15.7 | 3.9 |
| Sleep Physiology and Neurobiology | 23 | 11.7 | 2.9 |
| Hypersomnolence Disorders | 21 | 10.7 | 2.6 |
| Diagnostic Methods in Sleep Medicine | 19 | 9.6 | 2.4 |
| Secondary Sleep Disorders | 17 | 8.6 | 2.1 |
| **Total** | **197** | **100.0** | **24.6** |

# Supplementary Table S2. Sensitivity Analysis of Scoring Methodology: Performance Comparison Across Three Scoring Criteria

| **Model** | **Single-Try Accuracy (%)** | **Majority Accuracy (%)** | **Strict Accuracy (%)** | **Questions 3/3 Correct** | **Questions 2/3 Correct** | **Questions 1/3 Correct** | **Questions 0/3 Correct** | **Consistency Rate (%)** |
| --- | --- | --- | --- | --- | --- | --- | --- | --- |
| Gemini 2.5 Pro (Premium) | 97.5 | 95.9 | 95.9 | 189 | 0 | 3 | 5 | 98.5 |
| Claude Opus 4 (Premium) | 97.5 | 93.9 | 93.9 | 185 | 0 | 7 | 5 | 96.4 |
| ChatGPT GPT-4o (Premium) | 97 | 93.4 | 92.4 | 182 | 2 | 7 | 6 | 95.4 |
| Deepseek V3 (Free) | 91.9 | 91.4 | 91.4 | 180 | 0 | 1 | 16 | 99.5 |
| Claude 3.7 Sonnet (Previous) | 92.9 | 88.8 | 88.8 | 175 | 0 | 8 | 14 | 95.9 |
| Gemini 2.5 Flash (Free) | 90.4 | 88.3 | 88.3 | 174 | 0 | 4 | 19 | 98 |
| ChatGPT GPT-3.5 (Free) | 95.9 | 84.8 | 84.8 | 167 | 0 | 22 | 8 | 88.8 |
| xAI Grok3 (Free) | 85.8 | 81.2 | 81.2 | 160 | 0 | 9 | 28 | 95.4 |
| Llama 3 (Free) | 71.1 | 68.5 | 65.5 | 129 | 6 | 5 | 57 | 94.4 |
| Mean ± SD | 91.1 ± 8.5 | 87.4 ± 8.5 | 86.9 ± 9.2 |  |  |  |  | 95.8 |
| Range | 71.1–97.5 | 68.5–95.9 | 65.5–95.9 |  |  |  |  |  |
| Models ≥80% | 8/9 | 8/9 | 8/9 |  |  |  |  |  |

# Representative Sample Questions with Answer Keys

Selection Criteria and Rationale: The following eleven questions were selected to provide a representative sample of the complete 197-item question bank. Selection criteria ensured: (1) proportional representation across all seven AASM certification domains, with at least one question from each subdomain; (2) distribution of difficulty levels reflecting the overall question bank composition (approximately 30% basic recall, 50% application/analysis, and 20% complex clinical reasoning); (3) demonstration of the clinical vignette format and five-option multiple-choice structure consistent with AASM certification examination standards; (4) inclusion of one image-based question (Question 11) demonstrating polysomnographic interpretation skills. To protect examination security and maintain the validity of the assessment instrument for future research applications, the complete question bank is not publicly disclosed. Researchers with legitimate academic purposes may request access to the full dataset through formal data sharing agreements by contacting the corresponding author.

**Question 1. Sleep-Related Breathing Disorders**

A 52-year-old male with a BMI of 34 kg/m² presents with excessive daytime sleepiness and witnessed apneas. His polysomnography reveals an apnea-hypopnea index (AHI) of 45 events/hour with oxygen desaturation nadir of 78%. Which of the following is the most appropriate first-line treatment?

A) Positional therapy

B) Oral appliance therapy

C) Continuous positive airway pressure (CPAP)

D) Hypoglossal nerve stimulation

E) Uvulopalatopharyngoplasty (UPPP)

**Correct Answer: C**

**Question 2. Sleep Physiology and Neurobiology**

During which stage of sleep is the arousal threshold highest?

A) Stage N1

B) Stage N2

C) Stage N3

D) Stage R (REM)

E) Wake after sleep onset

**Correct Answer: C**

**Question 3. Diagnostic Methods in Sleep Medicine**

According to AASM scoring criteria, an obstructive apnea is defined as a ≥90% reduction in airflow lasting at least how many seconds?

A) 5 seconds

B) 10 seconds

C) 15 seconds

D) 20 seconds

E) 30 seconds

**Correct Answer: B**

**Question 4. Circadian Rhythm and Insomnia Disorders**

A 45-year-old female presents with chronic difficulty initiating and maintaining sleep for the past 8 months. She reports daytime fatigue but denies symptoms of depression. Which of the following is the first-line evidence-based treatment?

A) Zolpidem 10 mg at bedtime

B) Trazodone 50 mg at bedtime

C) Cognitive behavioral therapy for insomnia (CBT-I)

D) Melatonin 5 mg at bedtime

E) Diphenhydramine 25 mg at bedtime

**Correct Answer: C**

**Question 5. Hypersomnolence Disorders**

A 22-year-old male presents with excessive daytime sleepiness, cataplexy triggered by laughter, and hypnagogic hallucinations. His MSLT shows a mean sleep latency of 3.2 minutes with 3 sleep-onset REM periods. CSF hypocretin-1 level is 45 pg/mL (normal >110 pg/mL). What is the most likely diagnosis?

A) Idiopathic hypersomnia

B) Narcolepsy type 1

C) Narcolepsy type 2

D) Insufficient sleep syndrome

E) Kleine-Levin syndrome

**Correct Answer: B**

**Question 6. Circadian Rhythm and Insomnia Disorders**

A 19-year-old university student reports inability to fall asleep before 3:00 AM and difficulty waking for morning classes. He sleeps well on weekends until noon. Actigraphy confirms a delayed sleep-wake pattern. Which treatment combination is most appropriate?

A) Evening bright light therapy + morning melatonin

B) Morning bright light therapy + evening melatonin

C) Chronotherapy with progressive delay

D) Sedative-hypnotic medication at 10:00 PM

E) Stimulant medication in the morning

**Correct Answer: B**

**Question 7. Movement and Behavioral Disorders**

A 68-year-old male presents with a 3-year history of dream enactment behavior including punching and kicking during sleep. His wife reports he appears to be "fighting" during these episodes. PSG demonstrates REM sleep without atonia. Which condition is most strongly associated with this diagnosis?

A) Alzheimer's disease

B) Frontotemporal dementia

C) Parkinson's disease and Lewy body dementia

D) Vascular dementia

E) Normal pressure hydrocephalus

**Correct Answer: C**

**Question 8. Movement and Behavioral Disorders**

A 55-year-old female with restless legs syndrome has been treated with pramipexole 0.5 mg for 2 years. She now reports symptoms occurring earlier in the day, spreading to her arms, and requiring higher doses for relief. Her serum ferritin is 35 ng/mL. What is the most appropriate next step?

A) Increase pramipexole dose to 1.0 mg

B) Add ropinirole to current regimen

C) Iron supplementation and transition to alpha-2-delta ligand

D) Initiate opioid therapy

E) Refer for deep brain stimulation evaluation

**Correct Answer: C**

**Question 9. Secondary Sleep Disorders**

A 48-year-old female with congestive heart failure (EF 30%) undergoes polysomnography showing an AHI of 35 events/hour with predominantly central apneas demonstrating a crescendo-decrescendo pattern. What is the underlying breathing pattern?

A) Biot's respiration

B) Cheyne-Stokes breathing

C) Kussmaul breathing

D) Ataxic breathing

E) Paradoxical breathing

**Correct Answer: B**

**Question 10. Diagnostic Methods in Sleep Medicine**

Which of the following EEG findings is characteristic of Stage N2 sleep according to AASM criteria?

A) Alpha rhythm with eye blinks

B) K-complexes and sleep spindles

C) High-amplitude delta waves >75 μV

D) Low-amplitude mixed frequency with sawtooth waves

E) Vertex sharp waves only

**Correct Answer: B**

**Question 11. Sleep Physiology and Neurobiology**

*[See Supplementary Figure S1: 30-second polysomnography epoch]*

A 35-year-old male undergoes diagnostic polysomnography for evaluation of excessive daytime sleepiness. The accompanying figure shows a representative 30-second epoch from his sleep study. Based on these 30-second polysomnography epoch, which sleep stage is depicted in this epoch?

A) Stage W (Wake)

B) Stage N1

C) Stage N2

D) Stage N3

E) Stage R (REM)

**Correct Answer: E**


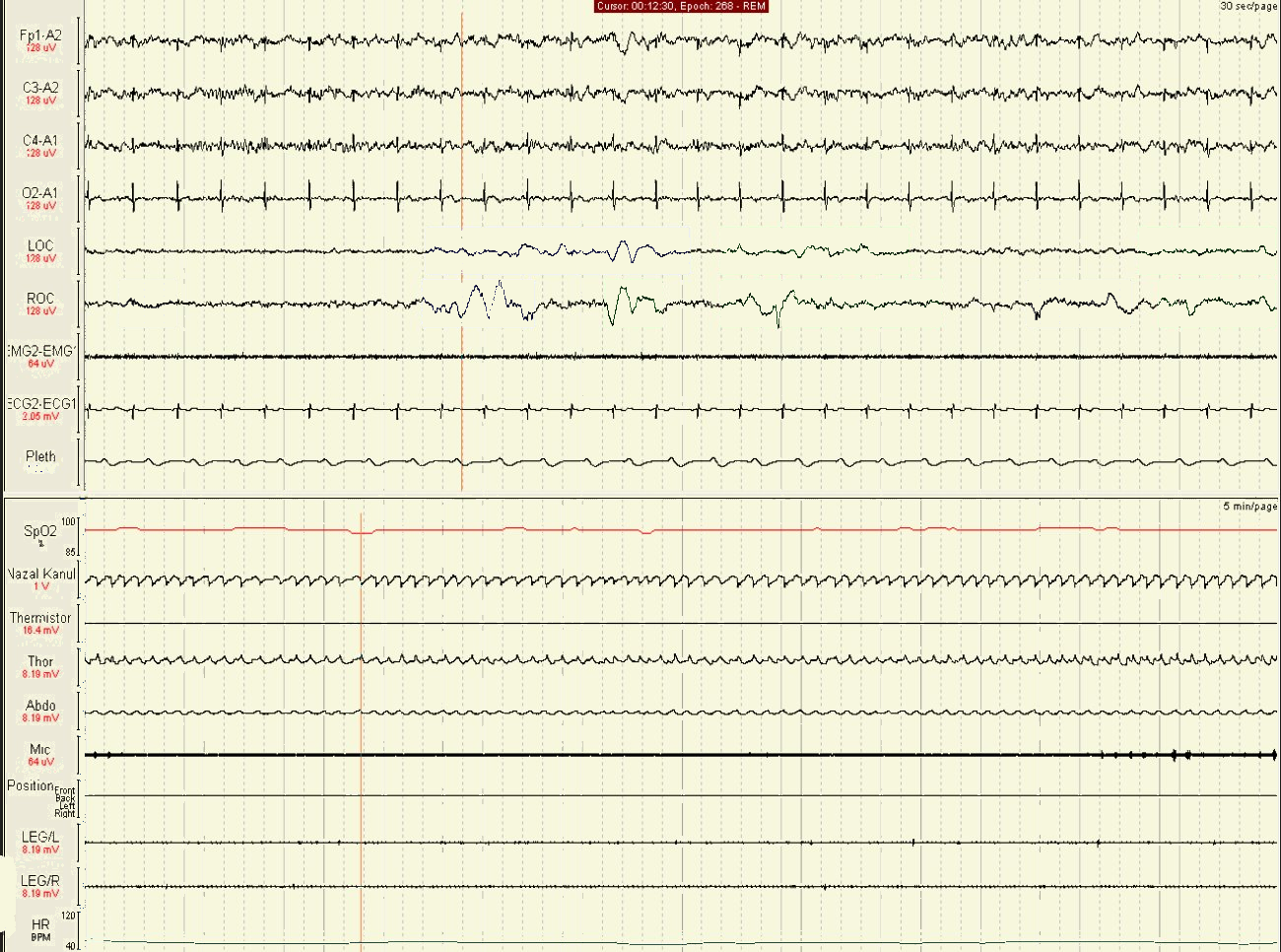


**Supplementary Figure S1.** Representative 30-second polysomnography epoch

*Abbreviations: AASM = American Academy of Sleep Medicine; AHI = Apnea-Hypopnea Index; BMI = Body Mass Index; CBT-I = Cognitive Behavioral Therapy for Insomnia; CPAP = Continuous Positive Airway Pressure; CSF = Cerebrospinal Fluid; EEG = Electroencephalogram; EF = Ejection Fraction; EOG = Electrooculogram; EMG = Electromyogram; MSLT = Multiple Sleep Latency Test; PSG = Polysomnography; REM = Rapid Eye Movement; RLS = Restless Legs Syndrome; UPPP = Uvulopalatopharyngoplasty.*

*Note: All questions are presented in multiple-choice format with five response options (A through E) consistent with the AASM certification examination structure.*
